# Articulating A Rights-Based Approach to HIV Treatment and Prevention Interventions

**DOI:** 10.2174/157016211798038588

**Published:** 2011-09

**Authors:** David Barr, Joseph J Amon, Michaela Clayton

**Affiliations:** 1 International Treatment Preparedness Coalition, New York, USA; 2 Health and Human Rights Division, Human Rights Watch, New York, USA; 3 AIDS and Rights Alliance for Southern Africa, Windhoek, Namibia

**Keywords:** HAART, highly active antiretroviral therapy, HIV prevention, human rights.

## Abstract

Since the beginning of the epidemic, the protection of human rights has been an integral component in the response to Human Immunodeficiency Virus (HIV). The high degree of stigma and discrimination associated with acquired immune deficiency syndrome (AIDS) has made human rights protection not only a priority to ensure the rights of people living with and at-risk for HIV, but to address public health goals as well. Advances in understanding the impact of antiretroviral treatment on HIV prevention provide exciting opportunities and even a paradigm shift in terms of AIDS prevention. However, this potential cannot be reached unless the advancement of human rights is a primary component of treatment and prevention programme and policy development. The use of antiretroviral treatment as prevention reinforces the value of basic principles related to the dignity and agency of people living with HIV to participate in the design and implementation of programmes, to be informed and to make informed decisions about their health and lives, to be protected from harm, and to have opportunities to seek redress and accountability for abuses. The possibility of using HIV treatment as a prevention tool means that now, more than ever, legal reform and community empowerment and mobilisation are necessary to realize the rights and health of people affected by HIV.

## INTRODUCTION

In 2001, United Nations (UN) member states agreed to goals that would provide HIV care, treatment and prevention services to all who need them [1]. The World Health Organization (WHO) *3 x 5* initiative [2] operationalised this goal which was reaffirmed by the June 2006 Political Declaration on HIV/AIDS [3], and unanimously adopted by the UN member states. At the same time, the Political Declaration recognized that combating HIV/AIDS is a pre-condition to achieving many of the Millennium Development Goals (MDGs) [4]. To complement these political commitments, funding mechanisms, such as the Global Fund to Fight AIDS, Tuberculosis and Malaria (Global Fund) and the United States (US) President’s Emergency Plan for AIDS Relief (PEPFAR) were created. Much has been accomplished: since that first agreement in 2001, more than five million people have gained access to antiretroviral therapy (ART), AIDS deaths and hospitalizations have decreased, and rates of new infections have been reduced in many countries [5]. In June 2011, the UN General Assembly (UNGA) reaffirmed its goals to address AIDS, setting ambitious targets for 2015 including the elimination of vertical HIV transmission, a 50% reduction in sexually-transmitted HIV infections and 15 million people on ART [6].

However, much remains to be done to fully address the global HIV pandemic and to meet 2011 UNGA and MDG targets by 2015. Under the revised WHO ART guidelines, nine million people in need of immediate treatment are not receiving it [6], and, despite some progress, rates of new infections continue to outpace rates of treatment delivery by almost two to one [7]. Competing global priorities and the global economic crisis have begun to divert commitments away from AIDS just as the effects of the investment in HIV prevention, treatment and care efforts are showing real results [8]. Increased financial and human resources are essential if MDG goals are to be met.

Insufficient resources for prevention and treatment programmes are not the only impediment to achieving universal access targets. Even when programmes are adequately funded, stigma, discrimination and a wide range of human rights abuses undermine HIV testing programmes and reduce their use, particularly among key vulnerable populations. This, in turn, impacts the ability of individuals to access and use ART where it is available. Furthermore, the effectiveness of many prevention interventions on behavioural change has proven insufficient to significantly stem the tide of new infections [9]. Furthermore, despite strong rhetorical commitment to recognising the link between human rights and HIV vulnerability, funding for rights-based interventions that explicitly target stigma and discrimination, human rights violations and structural impediments against people living with HIV/AIDS, women and other key affected populations is minimal [10].

In addition to its effectiveness in treatment, recent clinical studies have provided conclusive evidence of the impact of ART on the prevention of HIV transmission [11]. Most recently, the *HIV Prevention Trials Network* *(HPTN) 052* trial was stopped four years ahead of schedule because of the magnitude of the interim results. The study enrolled over 1750 serodiscordant couples in which the HIV-infected partner had between 350 – 550 CD4 cells/mm^3^. Half of the partners were randomized to start ART immediately. The other half delayed ART until their CD4 cell level fell to 250 cells/mm^3^ or they had an AIDS-defining illness. There were 27 new HIV infections in the untreated group and one new infection in the group that started treatment early, a 96% difference [12]. This research creates important new opportunities and public health, economic, and human rights arguments for expanded and improved integration of treatment and prevention efforts. The consequence of this understanding can be compared to historic turning-points in the past, including the development of:

- A test to detect HIV antibodies in 1985 [13],
- ART to prevent perinatal HIV transmission in 1991 [14], and
- Highly active ART to suppress viral replication, significantly reducing AIDS-related morbidity and mortality, in 1996 [15].

Each of these milestones, accompanied by vocal, and sometimes contentious, advocacy from scientists, activists and government officials, created a paradigm shift in how HIV policies and programmes were developed, prioritized, funded and implemented. The emerging evidence regarding the impact of ART on HIV transmission presents another opportunity for a powerful paradigm shift in the response to HIV that requires a careful examination of the scientific evidence, programmatic structure, and the important role of ensuring protection and promotion of human rights, including key principles of equity, empowerment and accountability.

A number of models, studies and initiatives have been formulated to operationalise “treatment as prevention” approaches, including the Joint UN Programme on HIV/AIDS (UNAIDS) *Treatment 2.0* Initiative [5], which seeks to optimise availability of a better fixed-dose antiretroviral combination, strengthen community-based service delivery and maximize the prevention impact of ART through rapid scale-up of treatment access to meet current ART guidelines. Mathematical models have proposed widespread population-based HIV testing and treatment, combined with expanded evidence-based HIV prevention programmes, which would lead to decreased HIV transmission to varying degrees [16]. In the US, a “test and treat” research study will test the viability of providing HIV testing to everyone in Washington, District of Columbia (DC) and the Bronx, New York and offer ART to all those with positive test results meeting current treatment guidelines (US guidelines recommend starting ART at <500 CD4 cells/mm^3^) [17].

The UNAIDS *Treatment 2.0* initiative provides the most rational framework for implementation of treatment as prevention approaches based on current information and available resources [5]. This approach seeks to scale up treatment access to provide coverage to all who meet current WHO treatment guidelines and reap the prevention impact as a secondary goal. This would provide time for additional research on the potential risks and benefits of earlier initiation of ART as well as time to determine better methods to increase and meet demand for HIV health services.

Some HIV treatment advocates have questioned the value of any discussion regarding potential implementation of treatment as prevention approaches while governments remain unable to meet current targets for treatment access [18]. However, integrated treatment as prevention approaches can prioritize those patients in greatest need of immediate treatment, while making a powerful argument for expansion by demonstrating long-term cost savings through reduced infection rates [19].

Some policy makers have also raised concerns that pursuit of treatment as prevention approaches will lead to further “medicalisation” of the HIV response and away from a commitment to other prevention efforts [20]. However, the potential impact of ART on prevention efforts provides greater impetus to increase access to voluntary HIV testing and improved linkage to care, and to build a diverse set of prevention interventions around treatment delivery services. At the same time, the need for rights-based approaches including community mobilization and community-based services – often at the centre of prevention efforts focused on behavioural change among key populations – are of primary importance if treatment as prevention is to realise its promise [21].

Successful HIV prevention and use of ART for either prevention or treatment both depend upon the ability of individuals and affected communities to seek out services and then use those services over the course of a lifetime. While increasing rates of HIV testing utilization is one important component, it is not the numbers of tests performed that, in and of itself, will lead to increased demand for and successful use of prevention and treatment services. HIV testing is not a goal, but a tool. It is a tool that can only be valuable if the conditions are in place to put the information one receives from testing to use. Those conditions include the availability of sustained and high-quality health-care and prevention services, including ART, along with policies and programmatic approaches that protect people from human rights abuses.

While testing is crucial, knowing one’s HIV status is often not enough. Even where treatment is free, countries have reported large gaps between numbers of people who test positive and those who start, and are able to maintain, treatment. Much more needs to be done to bridge the gap between testing and treatment, and keep people on treatment. There is still very little research on the factors that affect whether or not people choose to start treatment, but it is clear that the biomedical argument (regarding the benefit of ART in reducing morbidity and mortality) is not always sufficient motivation for people to start. This is even the case in wealthier settings: an analysis of 15 large cohorts consisting predominantly of North American and European patients found a wide range in baseline CD4 counts upon ART initiation [22].

An environment of safety in which people at risk for HIV can demand and use services without fear of stigma, discrimination and abuse of their human rights is a necessary prerequisite for implementation of all HIV treatment services, including those for prevention. Without sufficient human rights protection, seeking HIV services can often be more immediately dangerous than HIV itself. In any implementation of treatment as prevention, both top-down programmes including legal reform and ground-up empowerment and mobilisation programmes are essential to ensuring that the rights of HIV-affected people and communities are protected and to ensuring the long-term success and sustainability of these programmes.

## HUMAN RIGHTS ABUSES AS AN IMPEDIMENT TO TREATMENT AS PREVENTION GOALS

At the start of the HIV epidemic, the fear of an unknown and fatal disease led to public calls for mandatory testing and for a “right to know” others’ HIV status. Patients demanded the right to know the HIV status of their health care provider, and health care providers sought to know the status of their patients (with or without their knowledge or consent); mandatory HIV testing programmes were proposed, and often established, for a range of different groups, including pregnant women and infants, engaged couples, employees, students, immigrants, and sex offenders. Related to this demand for forced testing and disclosure were efforts to criminalise HIV transmission - punishing non-disclosure of status and, at times, even the failure to suspect one’s possible HIV status [23] - and to limit the movement of HIV-infected individuals [24].

Three decades later, despite greater understanding of HIV transmission, ignorance and stigma continue to drive discriminatory laws, policies and practices. Although offering little practical aid – either for individuals or for communities generally – laws criminalising HIV transmission and populations believed to be at high risk of HIV infection exist in more than 160 countries [25]. Since 2005, 14 African countries have passed HIV-specific laws that potentially criminalise all sexual behaviour among HIV-positive individuals, many of which even criminalise HIV transmission from women to their children [26]. These laws are often duplicative of existing provisions in the penal code, are contrary to international guidelines on HIV/AIDS and human rights, are difficult to enforce, and can have negative consequences for broader efforts to expand HIV testing and reduce stigma and discrimination^1^ [27].

The Uganda draft HIV/AIDS law introduced into Parliament in May 2010 is illustrative of how legal frameworks that fail to guarantee human rights can prove to be an impediment to efforts to expand HIV testing and put in place treatment as prevention programs. For example, the Uganda law provides that consent is not necessary for HIV testing when it is “unreasonably withheld” (section 12) and mandates routine testing for pregnant women and their partners, as well as for victims of sexual offenses ^2^ [28]. The bill also contains provisions requiring that a person convicted of drug use or possession of a hypodermic needle, charged with a sexual offense, or convicted of an offense involving prostitution, “be subjected to HIV testing for purposes of criminal proceedings and investigations”^3^ [28].

The Ugandan bill permits disclosure of HIV status without the consent of the person testing positive in several vaguely defined circumstances, including when, “in the opinion of the medical practitioner, [the HIV-positive person] poses a clear and present danger” to a person with whom he or she is “in close and continuous contact including but not limited to a sexual partner”^4^ [28]. Another provision of the bill imposes a “general penalty” of up to 10 years of imprisonment and/or a fine of no more than 4,800,000 Uganda shillings (United States [US]$2400), for any conduct that “contravenes the provisions of this Act” (section 45) [28]. Any person who fails to “take reasonable steps and precaution to protect him- or herself and others from HIV infection” is potentially subject to this penalty (section 3) [28].

At the same time that the HIV/AIDS bill was introduced, another bill aimed to make more severe existing penalties for homosexual sex (punishable by imprisonment for up to 14 years). The so-called “Anti-Homosexuality” bill mandated that anyone convicted of a homosexual act would face life imprisonment. Further, individuals convicted under the law who are HIV-positive would be subject to the death penalty, and all citizens would be required to report any “homosexual activity” to the police. Combined, the two bills present a challenge to effective HIV prevention and treatment efforts. Both bills were met with international condemnation and expired at the end of the parliamentary term without being voted upon; however, sponsors of both bills have pledged to re-introduce them.

The Ugandan bills, and other efforts to mandate HIV testing and disclosure (violating rights to privacy, confidentiality, autonomy, and non-discrimination) stand in sharp contrast with rights-based campaigns and “structural-rights” interventions [29]. These approaches focus on the right to health (information, access to testing and treatment, and other related socio-economic rights) linked to civil and political rights such as non-discrimination, the right to be protected from violence, and rights to speech and assembly. Rights-based approaches emphasize accountability and empowering vulnerable and socially marginalized populations, ensuring that HIV testing and treatment programmes exist, are accessible to all, of good quality, and link the knowledge gained by HIV testing to the ability to protect oneself or others and to access care and support [30].

Laws criminalising HIV transmission also frequently disparately criminalise women, who, as a result of pregnancy-related medical care, form the majority of those who know their HIV status, and may be unable to negotiate with sexual partners for safe sex because of fear of violence. While little quantitative evidence exists, qualitative research with diverse vulnerable populations frequently has found that fears of mandatory or coercive HIV testing and breaches in confidentiality drive individuals away from testing and treatment services.

Human Rights Watch explored the issue of domestic violence and women’s and children’s vulnerability to HIV in Uganda in 2002. One woman, Alice, described the outcome of her efforts to convince her husband to go with her to test for HIV: “He said, ‘if I know you’re positive I’m going to kill you.’ …I can’t even test the children because he’ll be angry and ask why” [31]. In June 2010, another Ugandan woman reported: “How will this [proposed HIV] law protect me? When my husband threw me out of the house, took away my property and my children, and tore my identity card and diplomas to pieces, the police, the courts, did nothing” [personal communication].

In China, drug users have reported that being tested for HIV was associated with being detained by the police and put into a drug detoxification or rehabilitation through labour (RTL) centre for as long as five years. Liu, a drug user in Guangxi province, told Human Rights Watch in July 2007: “I had been using drugs and decided to go get tested for HIV. I had just come from having my blood drawn on the CDC [Chinese government Centers for Disease Control] compound and police saw that my arm had an open mark and some blood. They stopped me and put me in detox.” Another drug user interviewed during the same month told us: “Sometimes I’m afraid I might be sick with AIDS but I’d rather be sick and free than go to get tested, get arrested, and be sick in detox or re-education through labour [RTL]” [32, 33]. In other cases, drug users are specifically excluded from ART programs, or told they must stop taking methadone (an essential component of their treatment for drug dependence) to access ART [34].

Men who have sex with men (MSM) are also often excluded from HIV prevention and treatment programmes because of discrimination and human rights abuses, including at the hands of medical providers and police [35]. Human Rights Watch documented the perspective of one man, Curtis, who said: “I try to keep myself healthy because if you go to the hospital, they won’t take care of you. If you got a bruise on your anus, that would make it worse. To be honest, if anything should happen to me, I am not going to the public hospital. I would buy over-the-counter medication or speak to my friends. I know that I am at risk but just to keep myself safe I cannot go to the hospital. Because if something should happen to me, I cannot go to the police because they will not help me” [36].

Internal and international migrants are another vulnerable population frequently denied access to HIV prevention and treatment [37, 38]. While in some countries, economic, social and cultural barriers result in the inaccessibility of care (and denial of the right to health) for migrants. In countries such as China and Russia, internal migrants who lack official residence status are often administratively ineligible to receive public health services. In other countries such as Botswana, refugees are excluded from accessing free government-provided treatment as non-nationals.

## ARTICULATING A RIGHTS-BASED APPROACH TO HIV PREVENTION AND TREATMENT DELIVERY

Over the past decade, proposals by public health authorities to increase HIV testing utilisation through routine testing (often with weakened informed consent procedures) [39] have been met with concern by human rights advocates seeking to protect rights to privacy, confidentiality and autonomy [40]. This debate has often been characterised - and caricatured - as one that pits human rights versus public health. The writers of this article, long-time human rights and AIDS treatment advocates, believe this is a false dichotomy. Without sufficient human rights protection, it is impossible to meet HIV public health goals, nor would those goals have any meaning. Conversely, the failure to meet public health goals represents a serious threat to the human rights of people affected by HIV. Successful responses to HIV depend upon articulation of models that drastically increase use of HIV testing, prevention, treatment and support services *and* do so in ways that foster human rights protection, reduce stigma and discrimination and encourage the sustained engagement of those directly affected by HIV.

The potential impact of ART as a prevention tool does not alter this approach. The expanded value of ART only heightens the need to find successful approaches to improved HIV service delivery *and* human rights protection. Before describing some parameters for rights-based approaches to HIV service delivery, a few basic principles should be outlined that can drive the development of attempts to realise the impact of ART on prevention:

- Guidelines determining the optimal time to start ART in the course of HIV disease must be based on what is best for the individual patient. People living with HIV should not be expected to begin therapy for the primary purpose of preventing HIV transmission. The primary purpose of treatment is treatment. Patients should not be compelled to risk earlier development of antiretroviral drug resistance and/or suffer drug-related side effects unless there is clear evidence that earlier use of ART can be beneficial for the patient in prolonging life and improving the quality of life.
- If resources are limited, decisions about who should receive ART must be based on the need to treat the sickest patients first and not based on perceived opportunities to prevent new infections. The best way to address this is to ensure that all those meeting current treatment guidelines have adequate access to ART and other health care services.
- The choice to use ART remains a personal choice. Patients have the right to decide not to take ART.
- The availability of second- and third-line treatment combinations is essential to long-term use of ART. This will be especially important as earlier treatment is considered to maximize both treatment and prevention benefits of ART.

As described above, of the estimated 15 million people living with HIV/AIDS who will likely need treatment in 2011, many face considerable stigma associated with HIV/AIDS, threats to their human rights, structural barriers to accessing medicine and health care services, and personal and social challenges in making and following through on HIV treatment decisions. A great deal of self-efficacy is often needed to navigate institutions providing HIV treatment and care, confront potential HIV stigma and discrimination, overcome gender-based barriers and structure one’s life to ensure success of HIV treatment, prevention and broader health interventions.

Much like universal access, the success of treatment as prevention approaches will depend as much on a well-defined, well-resourced and consistently applied strategy for meaningful involvement of people living with HIV (PLWH) and communities in designing, implementing and monitoring programs, as on funding. Such involvement needs to be ensured from the conception of programmes right up to the point of service delivery. The current reality, however, is far from this – while the greater involvement of people living with HIV (GIPA) has received much lip service, involvement of PLWH and communities continues to be minimal, conditional or even tokenistic.

Starting from the highest levels of health governance, there needs to be a paradigm shift regarding the role of patients and communities in delivering health services: rather than being viewed as subjects of interventions, they should be seen as priority collaborators, and interventions should be implemented in a true spirit of partnership that recognizes an individual’s ownership and ultimate decision-making power over his or her own health. However, the onus is on civil society, and more particularly on PLWH and communities who are particularly vulnerable to HIV infection, to clearly define what this requires in terms of concrete policy and programmatic actions.

Additionally, as pointed out by the Global Network of People Living with HIV (GNP+) at the WHO consultation on treatment as prevention, “the concept of ‘universal’ voluntary HIV testing cannot be achieved without major changes in both policy and social conditions… throughout the HIV epidemic, funding and political support have gone to easily measurable outcomes such as testing and treatment, and not to support social, legal and economic protection” [18]. Unless there is a concerted effort to alter this historical tendency, we are likely to repeat and amplify the negative consequences of treatment scale-up without also ensuring that social, economic and legal protections are in place. Following the principles articulated above, establishing a rights-based approach to HIV treatment as prevention service delivery will require a combination of policy development at national and global levels and grassroots programming.

## POLICY DEVELOPMENT: LAW, POLICY AND PRACTICE REFORM

The goal of law, policy, and practice reform is to create an enabling and non-discriminatory environment in which a legal and policy framework ensures respect for and protection of the right to health (including the right to information, access to testing and treatment, and other related socio-economic rights), as well as civil and political rights such as autonomy, privacy, the right to be protected from violence, rights to speech and assembly, and access to justice for PLWH and people vulnerable to HIV infection.

Such policy development should focus on the reform and monitoring of laws that impact the prevention of HIV transmission and the provision of care for those infected. These programmes may include audits of current laws and policies and their impact on the HIV response, and advocacy for reform of laws that can act as barriers to access to HIV prevention and treatment services, such as those which criminalise same sex relationships, sex work and drug use. Such interventions may also include advocacy for the enactment of laws that guarantee confidentiality of health information, including HIV status, and laws that prohibit discrimination on the basis of HIV or other status [41].

Legal reform programmes should also focus on the improvement of access to justice for people whose rights have been violated, and could include support for the establishment of human rights commissions, user-friendly courts or alternative dispute resolution mechanisms and for the provision of legal services for PLWH and members of affected and/or marginalized groups (women, young people, care-givers, survivors of sexual violence, orphans and vulnerable children, injecting drug users, sex workers, MSM, migrants and prisoners). These services could include legal advice and representation, strategic litigation, legal information and referral (including by phone), or assistance with informal or traditional legal systems (e.g. village courts). Legal services programmes could also include, or be linked to, community legal education; education of lawyers, judiciary and police; use of paralegals, volunteers, students and peer educators; outreach in community settings and in prisons; and monitoring, documentation of and advocacy for law reform [41].

Human rights training for health care workers focusing on informed consent, confidentiality, non-discrimination, duty to treat, and universal precautions is essential, as is the training and sensitisation of law enforcement agents on HIV and the human rights of vulnerable populations, particularly in terms of supporting access to services, non-discrimination, non-violence, and freedom from harassment, arbitrary arrest and detention.

It is also important to put in place programmes to promote the rights of women in the context of HIV. These programmes include interventions to change laws, policies and practices that discriminate against women. Examples of the harmful laws and practices affecting women and girls that have the potential to exacerbate their vulnerability to HIV include laws which restrict women’s economic opportunities, property and inheritance rights, inadequately criminalize or punish violence against women, and perpetuate harmful and inequitable gender norms.

In addition to law and policy reform, programmes to reduce stigma and discrimination are essential for the creation of a social environment that facilitates access to prevention and treatment services. Programmes to reduce stigma and discrimination should address their underlying causes—ignorance, fear, myths, social judgment, and lack of interaction with PLWH.

## GRASSROOTS APPROACHES: COMMUNITY MOBILISATION AND EMPOWERMENT

As important as policy developments aimed at improving the legal and policy environment, however, are those that promote change from the ground up. These programmes should focus on empowering those affected by HIV to know their rights in the context of the epidemic and draw them to formulate concrete demands for access to services and non-discrimination on the basis of HIV and other social status. In the context of treatment for prevention, it is of particular importance that individuals and communities are empowered and mobilised to claim their health-related rights. Empowerment in this context also means having the information and support necessary to make and follow through on treatment and prevention decisions and implement behaviour change to improve health.

### Empowerment:

In order to establish successful HIV prevention and treatment interventions, it is also important to provide services which strengthen the ability of individuals and communities to become invested in HIV care through empowerment approaches. Empowerment refers to the process through which individuals, organizations and/or communities gain control over “the planning and implementation of solutions to individually and locally felt problems, typically by decentralizing decision-making authority” [42]. Individual empowerment often refers to one’s own personal sense of competence or self-esteem. Organisational empowerment can include groups where individuals collaborate to share knowledge and experiences to raise their critical consciousness. Community empowerment refers to social and political activities in which individuals or groups participate. One commentator has suggested that “empowerment is…easier understood by its absence: powerlessness, helplessness, hopelessness, alienation, victimization, subordination, oppression, paternalism, loss of a sense of control over one’s life and dependency” [43].

The role of empowerment in community health is well-defined in social science literature^5^ [44]. Several studies show the effectiveness of empowerment techniques on various patient outcomes, including patient satisfaction, adherence to therapy and functional status [43]. These are not new concepts in the global response to HIV/AIDS. The GIPA principle (broadly interpreted to also include those at-risk of infection and those impacted by HIV in their communities), defined in the 1980’s and applied successfully embodies the empowerment model as an important, proven strategy to improve public health [45].

### Mobilisation:

Mobilisation refers here to the processes and outcomes of building local, community-based and peer-based networks and organisations as a mechanism to support treatment preparedness. These networks include advocacy, education and support programmes that value the role and participation of PLWH, and provide people with opportunities to be useful and valuable in their communities, to become role models for other HIV-positive individuals, and to show their communities that PLWH should be respected, not feared.

Mobilisation is not a new strategy in the global HIV/AIDS treatment effort. From the beginning of the HIV epidemic, much of the advocacy for HIV treatment access and education has involved mobilisation of new organisations and networks. Treatment advocacy and education requires a particular and specific expertise that can transcend the focus and capacity of many international HIV-focused non-governmental organisations (NGOs) and networks. In many cases, HIV treatment advocates have successfully created national, regional and international collaborations based on their commitment to this specific and important area of work, sharing their expertise and working in solidarity.

Empowerment and mobilisation approaches to service delivery are essential to the success of expanded treatment access and prevention efforts, including treatment for prevention. Legal protection alone will not do away with stigma. Ultimately, the reduction of stigma will depend on the willingness and ability of individuals to seek out health services. Service provision based on principles of community mobilisation and empowerment can provide the sustained engagement in care necessary for a lifetime of adherence.

Models for services espousing principles of empowerment and mobilisation abound. One example is the HIV Collaborative Fund, a project of the International Treatment Preparedness Coalition (ITPC), which has provided financial support to over 1000 community organisations in more than 70 countries for such services since 2004. The following are examples from reports of one-year programmes funded through the Collaborative Fund. Each of these programs received grants of $10,000 or less for this work [46].

- *AIDS Care China,* Guangxi, China - The Red Ribbon Center, a collaboration between Guangxi Longtan Hospital and AIDS Care China Guangxi, supported HIV treatment counselling for 661 PLWH (383 in-patients and 278 out-patients), telephone follow-up for patients on HIV treatment to support adherence, and supportive activities such as a summer camp held for 31 HIV-positive children and their family members. In total, 1540 PLWH (517 women and 1023 men) have started HIV treatment since 2007, and the Red Ribbon Center has provided HIV treatment counselling to 1115 people including PLWH, family members, out-patients, in-patients and children. Telephone follow-up has increased the patient return rate to the hospital from 40 percent to 75 percent, and has improved patients’ trust and experience of HIV treatment.
- *Positive Network of Mizoram (PNM),* Aizawl, Mizoram, India - PNM launched the first-ever HIV treatment education campaign in the northeastern Indian state of Mizoram, providing treatment education training for 120 participants (including PLWH, healthcare providers, government personnel, and other stakeholders), treatment advocacy training for 160 participants, and publication of HIV treatment information in a local newspaper to reach many hundreds of people. These activities have increased the involvement of PLWH in public discussions about HIV treatment access, and have enhanced the ability of PNM to monitor and advocate for supplies of ART and opportunistic infection drugs in Mizoram state.
- *Humanitarian Action,* St. Petersburg, Russia - Humanitarian Action worked with health professionals at the Botkin Hospital, the City Narcological Hospital, at rehabilitation centres, and in a new outpatient 'confidential' doctor network, to train and support providers in a realistic low-threshold model for maintaining adherence to long-term treatment in PLWH and injection drug users (IDU) that includes case management, addressing addiction and social problems, and providing all providers with case management information. In parallel, PLWH were trained as peer counselors at the Botkin Hospital about ART, counselling skills, and case management. A formal network of private 'confidential' doctors was created. Using a low-threshold multifaceted model for HIV treatment engagement and support, health care providers then reached 439 PLWH with offers of case management, provided 310 PLWH with a total of 654 medical consultations, and provided 402 PLWH with social and psychological care. Importantly, at least 96 PLWH from hard-to-reach populations - i.e. who had never previously contacted the system of health care institutions - were identified as a result of outreach work, including through prison outreach and through the use of a mobile bus, passed complete clinical and laboratory monitoring / examination and training in adherence, and currently receive ART.
- *Women Together Support Organisation,* Emkhuzweni, Swaziland - This project organized HIV-positive women and their communities in Mvembili and the Timphisini Inkhundla, with linkages to care at three health centres. The project began by conducting an HIV treatment education workshop for 60 HIV-positive women and then held an HIV/tuberculosis (TB) training of trainers for 30 HIV-positive women. With these trained HIV-positive women, the organisation launched an HIV/TB treatment campaign that reached 1500 people. Treatment supporters who are HIV-positive women are trained to help on adherence counselling and ensure those on treatment take medication correctly; community members have come to embrace and support PLWH in the communities (as evidenced by the number of people who come for information on how to help and sort out problems with family members who are on treatment). Women living with HIV report being more empowered on adherence to their treatment and better able to deal with the side effects of antiretrovirals. Women continue to ask for specific materials about treatment (for example, women ask about ART and menstruation and menopause), two cases of cervical cancer have been discovered, and women feel empowered to report stigma related to treatment.
- *CHECCOS,* Guadalajara, Mexico - In Mexico, the group CHECCOS supported advocate leadership development, aiming to empower PLWH in the four Mexican Social Security Institute Regional Hospitals from Guadalajara's metropolitan area, which care for patients of the 136 municipalities (2170 patients), as well as two civil hospitals. CHECCOS monitored HIV health provision at 50 hospitals throughout the metropolitan region. Using a database with e-mails from PLWH to record any shortages or abnormalities in care, the project was able to identify and report on 17 drugs in shortage or stock-outs, 16 instances of shortages of HIV monitoring tests, and three reports of poor compliance with medical protocols. Twenty-six PLWH dared to directly report shortages and face public servants, and all of them solved their need for treatment.
- *Family Support and Orphan Care for PLWHA (FSOCHA), *Jinja, Uganda - The project trained 30 FSOCHA members in HIV treatment literacy, conducted initial community trainings and conducted an HIV treatment literacy training for 32 PLWH and community AIDS workers. The peer counsellors were then supported by follow-up meetings, printed educational materials to distribute (more than 3430 copies), radio advertising and interviews, and community-wide meetings for treatment education and sensitisation (involving more than 200 people). The project was able to document a range of initial outcomes: clients in all three sub-counties reported a notable increase in level of treatment adherence; increased numbers of PLWH seeking cotrimoxazole (Septrin) for prevention of opportunistic infections in Buyengo; increased knowledge about treatment centre options so that members can seek services elsewhere when having problems with certain centres; and commitments by community leaders to write to service provides to address key issues.

These examples are just a few of the many programmes developed and run by community-based organisations often led by people living with HIV/AIDS. For relatively small amounts of money – in the examples cited above, each organisation received approximately US$10,000 a year - these programs are able to engage hard-to-reach key populations in HIV prevention, testing and care, showing tangible results such as:

- Improved treatment adherence
- Improved uptake of HIV testing utilisation
- Reduction of stigma and discrimination
- Strengthened linkages to drug treatment, sexual and reproductive health services, tuberculosis (TB) care and other essential health services
- Strengthened linkages to advocacy and monitoring at national and global levels

Despite evaluations showing the effectiveness of such approaches in both reaching key populations and providing them with effective services [47], funding remains insufficient. In its Five-Year Evaluation, the Global Fund was unable to provide information about support flowing directly to community-based organizations [48]. The Global Fund has responded to this need through targeted funding for community systems strengthening and for key affected HIV populations. However, the results of these efforts remain unevaluated. The large majority of bi- and multi-lateral resources for the HIV response are provided to national governments or to large international NGOs. It is difficult and often impossible for community-based organizations representing key populations to access these funds. Yet, ultimately, scale-up of community-based and -led approaches that protect and advance human rights for HIV-infected and vulnerable populations may be the most efficient and effective way to provide HIV health and support services in ways that can maximize the benefits of ART as both treatment and prevention.

## CONCLUSIONS

Since the beginning of the epidemic, guaranteeing human rights has been an essential aspect of successful HIV/AIDS programmes. The potential of treatment as prevention provides exciting opportunities and even a paradigm shift in terms of AIDS prevention. However, this potential cannot be reached unless respect, protection, and advancement of human rights are primary components of treatment and prevention programme and policy development. The potential of treatment as prevention does not fundamentally change basic principles related to the dignity and agency of people living with HIV to participate in the design and implementation of programmes, to be informed and to make informed decisions about their health and lives, to be protected from harm, and to have opportunities to seek redress and accountability for abuses. The introduction of the possibility of HIV treatment as prevention means that now, more than ever, top-down legal reform and ground-up empowerment and mobilisation are necessary to realise the rights and health of people affected by HIV.

## Footnotes

1 There is no evidence that using the criminal law to respond to HIV is effective in protecting public health, and some evidence that it may in fact cause harm. See, for example, Burris *et al*. [27].

2 “The following persons shall be subjected to routine HIV test for purposes of prevention of HIV transmission (a) the victim of a sexual offence; (b) a pregnant woman; (c) a partner of a pregnant woman.” [28, section 14]

3 “The following persons shall be subjected to HIV testing for purposes of criminal proceedings and investigations where (a) a person is convicted of drug abuse or possession of hypodermic instrument associated with drug abuse; (b) a person is charged with a sexual offence; (c) a person is convicted of an offence involving lewdness” [28, section 13]

4 "Notwithstanding section 19 a person may disclose information concerning the result of an HIV test or related medical assessments of a person tested, under the following circumstances . . . (f) [to] any other person with whom an HIV infected person is in close and continuous contact including but not limited to a sexual partner, if the nature of contact, in the opinion of the medial [sic] practitioner, poses a clear and present danger of HIV transmission to that person." [28, section 21]

5 For an excellent overview of the literature of empowerment theory and research, see Perkins and Zimmerman [44].

## ABBREVIATIONS

AIDS: = Acquired Immune Deficiency Syndrome
ART: = Antiretroviral Therapy
CDC: = (United States) Centers for Disease Control and Prevention
DC: = District of Columbia
FSOCHA: = Family Support and Orphan Care for PLWHA
GIPA: = Greater Involvement of People Living with HIV
GNP+: = Global Network of People Living with HIV
HAART: = Highly Active Antiretroviral Therapy
HIV: = Human Immunodeficiency Virus
HPTN: = HIV Prevention Trials Network
IDU: = Injection Drug User(s)
ITPC: = International Treatment Preparedness Coalition
MDGs: = Millennium Development Goals
MSM: = Men Who Have Sex with Men
NGO: = Non-governmental Organisation
NY: = New York
PEPFAR: = United States President’s Emergency Plan for AIDS Relief
PLWH: = People Living With HIV
PNM: = Positive network of Mizoram
RTL: = Rehabilitation (or Re-education) Through Labour
TB: = Tuberculosis
UN: = United Nations
UNAIDS: = Joint United Nations Programme on HIV/AIDS
UNGA: = United Nations General Assembly
US(A): = United States (of America)
WHO: = World Health Organization
